# The impact of childhood abuse on adult self-esteem and emotional regulation

**DOI:** 10.1080/07853890.2021.1896171

**Published:** 2021-09-28

**Authors:** André Pereira, José P. Santos, Pedro Sardinha, Jorge Cardoso, Catarina Ramos, Telma Almeida

**Affiliations:** a IUEM – Instituto Universitário Egas Moniz, Caparica, Portugal; b CiiEM – Centro de Investigação Interdisciplinar Egas Moniz, IUEM

## Abstract

**Introduction:**

The occurrence of traumatic experiences in childhood can lead to a disruption in the development of secure internal representations, with a negative impact on self-esteem [1]. In addition, child victimisation may compromise their emotional regulation skills [2] and it has been shown that individuals with lower self-esteem have less emotional regulation skills [3]. Studies with adults showed that those who suffered several types of abuse during childhood tend to have worse interpersonal relationships [4]. This research has the main goals, to identify the relationship between the impact of childhood abuse on adult self-esteem and emotional regulation, and the relationship between self-esteem and emotional regulation. This is the first study in a Portuguese sample that integrates those variables.

**Materials and methods:**

The study design is descriptive, observational, and cross-sectional. The sample was composed of 96 Portuguese adults (over 18 years old) (*M* = 27.95 years, *SD* = 11.60). The participants answered online to a sociodemographic questionnaire, Portuguese versions of the Childhood Trauma Questionnaire (CTQ) [5], the Difficulties in Emotion Regulation Scale (DERS) [6], and the Rosenberg Self-Esteem Scale (RSES) [7]. The link to the study was disclosed by e-mail and in social networks. The study was conducted in accordance with all the ethical principles.

**Results:**

We found significant statistical positive correlations between the total score of the CTQ and the total score of the DERS (*r* = 0.422, *p*<.001) and the sub-scales of DERS: the not acceptance of emotional responses (*r* = 0.311, *p*=.002), difficulties engaging in goal-directed behaviour (*r* = 0.243, *p*=.017), impulse control difficulties (*r* = 0.431, *p*<.001)), limited access to emotion regulation strategies (*r* = 0.465, *p*<.001), and lack of emotional clarity (*r* = 0.209, *p*=.041). Furthermore, we observed statistical negative correlations between the total score of RSES and the total score of the CTQ (*r*=–0.319, *p*=.002), the total score of the DERS (*r*=–0.561, *p*<.001) and the sub-scales of DERS: the not acceptance of emotional responses (*r*=–0.413, *p*<.001), difficulties engaging in goal-directed behaviour (*r*=–0.336, *p*=.001), impulse control difficulties (*r*=–0.373, *p*<.001), limited access to emotion regulation strategies (*r*=–0.508, *p*<.001), and lack of emotional clarity (*r* = −0.542, *p*<.001).

**Discussion and conclusions:**

In our study, traumatic experiences in childhood were associated with emotional regulation difficulties in adults, which is corroborated by other studies [2]. Additionally, we found that individuals who were victimised in childhood tend to have a perception of lower self-esteem in adult life. The relationship between child abuse and self-esteem and also between child abuse and the emotional regulation difficulties proves the need to develop psychological intervention aiming to enhance the positive self-esteem and the adaptative emotional skills in adulthood. This research highlights the importance of developing intervention programs in victimised children, to reduce the impact of victimisation on self-esteem and emotional regulation.
